# Non-Small Cell Lung Cancer with Epidermal Growth Factor Receptor (EGFR) Common Mutations: New Strategies

**DOI:** 10.3390/cancers17091515

**Published:** 2025-04-30

**Authors:** Brigida Stanzione, Alessandro Del Conte, Elisa Bertoli, Elisa De Carlo, Martina Bortolot, Sara Torresan, Michele Spina, Alessandra Bearz

**Affiliations:** 1 Department of Medical Oncology, Centro di Riferimento Oncologico di Aviano (CRO) IRCCS, 33081 Aviano, Italy; alessandro.delconte@cro.it (A.D.C.); elisa.bertoli@cro.it (E.B.); elisa.decarlo@cro.it (E.D.C.); martina.bortolot@cro.it (M.B.); sara.torresan@cro.it (S.T.); mspina@cro.it (M.S.); alessandra.bearz@cro.it (A.B.); 2 Department of Medicine (DME), University of Udine, 33100 Udine, Italy

**Keywords:** EGFR mutation, non-small cell lung cancer, target therapy

## Abstract

Epidermal growth factor receptor (EGFR) mutations are described in 10–15% of Caucasian patients and in 50% of Asian patients with non-squamous non-small cell lung cancer (NSCLC). The introduction of tyrosine kinase inhibitors (TKIs) has revolutionized the therapeutic scenario and has changed the natural history of the disease. Despite the results obtained with osimertinib, a third-generation TKI, most patients experience disease progression. The search for new therapeutic strategies both to enhance first-line treatment and to ensure adequate second-line therapies represents an unmet medical need, towards which all efforts are being concentrated. In this review, we describe the main strategies identified to improve the prognosis of patients with EGFR-mutated NSCLC.

## Simple Summary

The prognosis of non-small cell lung cancer with common EGFR mutations remains poor, despite the results obtained with the introduction of tyrosine kinase inhibitors. Disease progression during targeted therapy is linked to the development of resistance mechanisms. This literature review aims to summarize and clarify the main therapeutic strategies developed to address this important unmet medical need, including the latest updated and recently presented data.

## 1. Introduction

**Figure 1:**
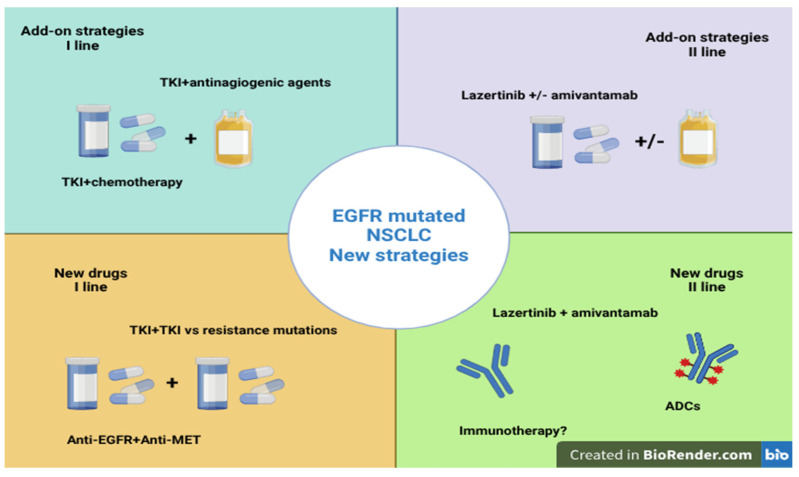
Main new strategies in EGFR-mutated NSCLC: novel approaches for EGFR-mutated NSCLC involve either using new therapies or improving existing ones by adding other drugs.

In 2022, lung cancer was the leading cause of cancer incidence and death worldwide [1]. It is typically associated with smoking habits, although its incidence is increasing in non-smokers, particularly women and young people. Epidermal growth factor receptor (EGFR) gene-activating mutations were first identified in non-small cell lung cancer (NSCLC) in 2004 [2]. These mutations are present in 10–15% of cases of non-squamous NSCLC in the Caucasian population but can reach an incidence of 50% in the Asian population [3]. They are usually found in non-smokers, with a higher incidence in young and female patients. EGFR mutations usually involve exons 18–21, which are part of the tyrosine kinase domain. So-called “common mutations”, including del19 and L858R in exon 21, representing about 90% of all EGFR mutations, have shown a marked sensitivity to tyrosine kinase inhibitors (TKIs), whose introduction into the medical armory has significantly changed the prognosis of these patients and the therapeutic approach to lung cancer [4]. Conversely, “uncommon mutations”, which constitute only 5–10% of cases, are represented by hundreds of mutations affecting exons 18–21 and are associated with a worse prognosis, showing extremely heterogeneous behaviors [5]. Among these, exon 20 insertion mutations, accounting for 4–12% of cases of EGFR-mutated NSCLC, are the most frequent and are described especially in the Asian population and in non-smoker patients and are characterized by a poor response (less than 10%) to TKIs [6]. Other uncommon mutations are represented by G719X (exon 18), S768I (exon 20), and L861Q (exon 21), described with a prevalence of ~3%, ~1%, and ~1%, respectively. The presence of compound mutations, the finding of which is increasingly frequent since the introduction of more sophisticated detection methods such as next-generation sequencing (NGS), can further modify the clinical history of the disease [7]. The quality of life and survival results of patients with advanced NSCLC with EGFR common mutations have been drastically altered by the approval of three generations of anti-EGFR tyrosine kinase inhibitors (TKIs) [8]. These medications are characterized by oral delivery and generally modest adverse effects (AEs), primarily cutaneous and gastrointestinal toxicity, providing acceptable management. Gefitinib and erlotinib are the first-generation anti-EGFR TKIs that were first studied in advanced non-small cell lung cancer and later, for the first time, outperformed chemotherapy in the first-line treatment of EGFR-mutated NSCLC [9]. Compared to first-generation TKIs, second-generation TKIs, afatinib and dacomitinib, have demonstrated a better EGFR inhibition because of their irreversible covalent binding mechanism [10,11,12,13]. Although they demonstrated their superiority compared to gefitinib in the LUX-Lung 3-6-7 studies and in the ARCHER study, respectively, achieving a progression-free survival (PFS) of 11 months for afatinib and 14.7 months for dacomitinib, their toxicity, poor handling, and lack of benefit to overall survival (OS) and the advent of osimertinib have severely limited their use in clinical practice. Osimertinib is a third-generation TKI with a better tolerability profile and strong antitumor activity by combining irreversibility with high selectivity over EGFR-mutated receptors [14]. Osimertinib was first used in clinical practice after progression to first- or second-generation TKIs, when resistance mechanisms developed, usually after 9–14 months of treatment. Specifically, in patients with the T790M mutation, it proved to be more beneficial than chemotherapy [15]. In the FLAURA trial, it showed superiority over first-generation EGFR TKIs in frontline treatment, with a median OS of 38.6 months (95% confidence interval [CI], 34.5 to 41.8) versus 31.8 months (hazard ratio (HR), 0.799; 95% CI, 0.641, 0.997), a median PFS of 18.9 months versus 10.2 months (HR, 0.46 [95% CI, 0.37, 0.57]), and an objective response rate (ORR) of 80% (95% CI, 75 to 85%) [16]. Osimertinib has demonstrated higher intracranial activity and a manageable safety profile. Although it is active against numerous resistance mutations that arise following first- and second-generation TKIs, the efficacy of osimertinib is also time-limited, since the onset of several types of resistance mechanisms is described [17]. In particular, the study of resistance mechanisms has allowed us to distinguish three different categories: on-target mechanisms, related to intrinsic additional mutations of EGFR; off-target mechanisms, related to other intracellular pathways [such as the mitogen-activated protein kinase (MAPK) pathway or Phosphoinositide 3-kinase (PI3K)-mammalian target of rapamycin (mTOR) pathway]; and histological transformation [18]. To understand the biological rationale underlying disease progression and to plan the subsequent treatment, the molecular study of the disease through a tissue re-biopsy and/or a liquid biopsy is essential. Tissue re-biopsy, in particular, is indispensable to document histological transformation. Among on-target mechanisms, the T790M mutation is well known: it is a typical mechanism of acquired resistance to first- and second-generation TKIs, being described in up to 50% of cases [19]. This mutation determines a steric conformation that prevents the binding of first- and second-generation TKIs, but not of osimertinib. Other on-target mechanisms are represented by EGFR amplification or by the acquisition of further point mutations such as C797S [20]. Regarding off-target mechanisms, another common resistance mechanism to EGFR-TKIs is the activation of alternative pathways that circumvent EGFR inhibition, such as the Ras–Raf–MEK–ERK cascade or MET amplification (10–15% of cases), which activates the downstream AKT pathway, the crucial signaling pathway for antiapoptosis and cell proliferation [21]. About 2% of cases of resistance to osimertinib and 12% of cases of resistance to first- and second-generation EGFR-TKIs have been identified to have ErbB2 gene amplification [22]. Furthermore, mutations in FGFR, BRAF, KRAS, and PIK3CA may result in resistance to first-, second- and third-generation EGFR-TKIs, giving cancer cells an edge in survival [23]. The possibility to associate to osimertinib a target therapy against these resistance mechanisms can somehow reverse resistance or, in any case, act on intratumoral heterogeneity [24]. Finally, about 2–15% of patients treated with EGFR-TKIs develop a histological transformation from adenocarcinoma to small cell lung cancer (SCLC), or rarely to squamous carcinoma, acquiring resistance to EGFR-TKIs. The transformation into SCLC can be linked to the co-presence of mutations in tumor suppressor genes, such as RB1 or TP53, or to the presence, since the onset of the disease, of different cell clones [25,26,27]. Thus, the emergence of resistance mechanisms poses a significant challenge in clinical practice. For this reason, finding effective second-line therapies and bolstering first-line treatment are also significant research topics (Figure 1). In this literature review, we summarize the main therapeutic strategies developed to improve the prognosis of patients with EGFR-mutated NSCLC, reporting the results of the most significant studies as well as the updated data that have been recently presented. 

## 2. Update on Advanced NSCLC with EGFR Common Mutations

### 2.1. Strengthening the First-Line Treatment

#### 2.1.1. Combination of TKIs with Chemotherapy

Despite the demonstrated efficacy of osimertinib and its widespread use in clinical practice, most patients experience disease progression [28,29]. One of the mechanisms identified to enhance first-line treatment efficacy and to postpone the onset of resistance mechanisms is the association between osimertinib and chemotherapy (CT). The rationale for this therapeutic option is to combat intratumoral heterogeneity by exploiting a double mechanism of action. Updated data relating to this topic have recently been presented. Several studies have evaluated the effectiveness of the association between chemotherapy and TKIs with different schedules: administered concurrently or sequentially, as maintenance therapy after chemotherapy induction, or intercalated. The most significant results were obtained by simultaneously administering TKI and chemotherapy (Table 1). A recent meta-analysis analyzed four trials, including 1413 patients [30,31,32,33,34]. When EGFR-TKI and chemotherapy were combined, the PFS was considerably higher than when EGFR-TKI was used alone (HR: 0.52 [95%CI: 0.45–0.59]; *p* < 0.00001). All examined subgroups showed the PFS benefit, but patients with brain metastases showed the greatest benefit (HR: 0.41 [95% CI: 0.33–0.51]; *p* < 0.00001) when compared to those without (HR: 0.57 [95% CI: 0.44–0.74]; *p* < 0.0001). Even in the subgroup of patients with ECOG PS 2, which were included in only one trial [32], PFS was better with the combination. Additionally, the combination showed an advantage in terms of OS (HR:0.69 [95% CI: 0.52–0.93]; *p* = 0.01) and ORR (HR: 1.93 [95% CI: 1.50–2.47]; *p* < 0.00001) [30]. FLAURA2, an international phase III study, enrolled 557 patients worldwide, confirming data in a non-Asian population [34]. In terms of safety, the combination was up to five times more likely to cause grade 3 or higher adverse effects than EGFR-TKI alone. The primary toxicities were digestive (mucositis, nausea, vomiting, and lack of appetite), hematological (anemia and neutropenia), and fatigue/asthenia, typically more associated with chemotherapy.

#### 2.1.2. Combination of TKIs with Antiangiogenic Agents

According to preclinical research, the EGFR and VEGF (vascular endothelial growth factor) signaling pathways work together to promote tumor growth [35]. Based on clinical research, VEGF expression is linked to increased vascularization in NSCLC, and overexpression of VEGF is associated with a poor prognosis in NSCLC. Patients with EGFR-mutated NSCLC have significantly longer OS when their serum VEGF levels are lower (HR 0.277, *p* = 0.018), and those who have lower serum VEGF levels after treatment have better PFS. As a result, the association of antiangiogenic agents with EGFR TKIs has been assessed in a number of randomized controlled clinical trials (Table 2).

The combination of bevacizumab with first-generation EGFR TKIs has been shown to be able to achieve consistent improvements in PFS but typically no effect on OS. In a meta-analysis published by Zhang et al., seven trials with a total of 1612 patients were analyzed [36,37,38,39,40,41]. Regardless of the type of EGFR mutation or the existence of brain metastases, TKIs in combination with antiangiogenic agents significantly improved ORR (HR, 1.10; 95% CI, 1.01–1.20; *p* = 0.029) and PFS. Specifically, HR of PFS was 0.58 (95% CI: 0.48–0.71; *p* < 0.001) in the subgroup with the L858R mutation. The OS following combination treatment was comparable to that after TKI monotherapy. TKIs combined with antiangiogenic drugs showed a markedly greater incidence of grade 3 or higher adverse events in the safety studies. The addition of bevacizumab to osimertinib, on the other hand, has been inconsistent in improving PFS. According to the ETOP meta-analysis assessing the role of bevacizumab added to EGFR TKIs, smoking populations would benefit more than non-smoking ones [42]. In comparison to erlotinib monotherapy, the RELAY phase III study evaluated the combination of erlotinib and ramucirumab (anti-VEGFR2) and found a significant PFS benefit with a median of 19.4 months and HR 0.59 (95% CI, 0.46–0.76), but no OS benefit [40].

**Table 2:** Main prospective clinical trials of combination of TKI and antiangiogenic agents.

Clinical Trial	Phase	Treatment	Median PFS	Median OS
Seto et al. [37]	2	Erlotinib + bevacizumab vs. Erlotinib	16 months 9 months	47 months 47.4 months
Stinchcombe et al. [38]	2	Erlotinib + bevacizumab vs. Erlotinib	17.9 months 13.5 months	32.4 months 50.6 months
Saito et al. [39]	3	Erlotinib + bevacizumab vs. Erlotinib	16.9 months 13.3 months	-
Nakagawa et al. [40]	3	Erlotinib + ramucirunab vs. Erlotinib	19.4 months 12.4 months	-
Zhou et al. [41]	3	Erlotinib + bevacizumab vs. Erlotinib	18 months 11.3 months	-

PFS: progression-free survival; OS: overall survival.

#### 2.1.3. New Third-Generation TKIs

Among the new third-generation TKIs, lazertinib deserves a special mention. It is an irreversible EGFR inhibitor, with similar efficacy to osimertinib in terms of ORR and survival, but with a greater ability to cross the blood–brain barrier, ensuring better intracranial response rates. In addition to the efficacy data, reassuring safety data have emerged from the LASER201 and, more recently, LASER301 studies: in fact, lazertinib is characterized by less toxicity at the skin and cardiological level, with less impact on QT [43,44]. In LASER301, lazertinib significantly extended median PFS across 393 patients compared to gefitinib (20.6 vs. 9.7 months; hazard ratio [HR], 0.45; 95% CI, 0.34 to 0.58; *p* < 0.001) [44]. In both groups, the ORR was 76% (odds ratio: 0.99; 95% CI: 0.62 to 1.59). With lazertinib, the median duration of response (mDOR) was 19.4 months (95% CI: 16.6 to 24.9) vs. 8.3 months with gefitinib (95% CI: 6.9 to 10.9). At the interim analysis, the overall survival data were immature. Lazertinib and gefitinib had 18-month survival rates of 80% and 72%, respectively (HR, 0.74; 95% CI, 0.51 to 1.08; *p* = 0.116).

Aumolertinib, another third-generation EGFR-TKI, was evaluated originally after disease progression in patients with locally advanced or metastatic NSCLC who developed an EGFR T790M mutation after progression on first- or second-generation EGFR TKI therapy, in the APOLLO trial [45]. Positive results have also been obtained from phase III research (AENEAS) that compared the safety and effectiveness of aumolertinib to gefitinib, as a first-line treatment for patients with locally progressed or metastatic NSCLC with EGFR mutations [46]. Compared to gefitinib, aumolertinib significantly prolonged PFS (hazard ratio, 0.46; 95% CI, 0.36 to 0.60; *p* < 0.0001). In contrast to 9.9 months with gefitinib (95% CI, 8.3 to 12.6), the median PFS with aumolertinib was 19.3 months (95% CI, 17.8 to 20.8). In the aumolertinib and gefitinib groups, the ORR and disease control rate (DCR) were comparable (73.8% and 72.1%, respectively; 93.0% and 96.7%, respectively). With aumolertinib, mDoR was 18.1 months (95% CI, 15.2 to not applicable), while with gefitinib, it was 8.3 months (95% CI, 6.9 to 11.1). Patients receiving aumolertinib experienced rash and diarrhea (any grade) in 23.4% and 16.4% of cases, respectively, compared to 41.4% and 35.8% of patients receiving gefitinib. In untreated EGFR-mutant patients with advanced NSCLC, a combination trial of aumolertinib and anlotinib, an oral multitarget antiangiogenic TKI, showed markedly elevated toxicity, indicating that the combined therapy approach was an unacceptable therapeutic decision in this context [47].

Furmonertinib is an oral, once-daily, and widely effective mutant-selective EGFR TKI that targets both common and rare mutations, such as EGFR exon 20 insertion mutations. In the FURLONG trial, furmonertinib showed improved efficacy over gefitinib, when used as a first-line treatment for Chinese patients with EGFR-mutant NSCLC, with an acceptable safety profile, with a PFS of 20.8 months (95% CI 17.8–23.5) in the furmonertinib group and 11.1 months (9.7–12.5) in the gefitinib group (HR 0.44, 95% CI 0.34–0.58; *p* < 0.0001) [48].

#### 2.1.4. Fourth-Generation TKIs

Among the main mechanisms of on-target resistance to third-generation TKIs, the appearance of a tertiary point mutation such as C797S or the loss of T790M are widely described. In particular, C797S, accountable for about 40% of occurrences of drug resistance, prevent inhibitors from forming a covalent link with the cysteine residue at position 797 on the border of the ATP-binding pocket. It has been demonstrated that when the C797S and T790M mutations are cis (located at the same allele), lung cancer cells are resistant to both the first- and third-generation EGFR-TKIs. However, when the C797S and T790M mutations are trans (located in distinct alleles), lung cancer cells have been demonstrated to respond well to the combined therapy of first- and third-generation EGFR-TKIs [49,50]. Fourth-generation TKIs were developed with the aim of overcoming resistance to third-generation TKIs and to act on the most frequent tertiary mutations, such as C797S.

Most of the studies on the new fourth-generation TKIs are still at an early stage; in some cases, the development of these drugs has suffered a setback, such as for BLU-701 and BLU-945 [51]. BDTX-1535, actually in a phase I/II dose expansion study, has demonstrated to be active in NSCLC patients across nearly all EGFR mutations, including T790M, C797S, L747P, L718Q, and compound mutations [52]. In a highly pre-treated population of 27 NSCLC patients, the ORR was 36%, with the majority of patients exhibiting sustained responses [53,54]. The most frequent side effects were diarrhea (35%, no grade ≥3 observed) and rash (70%, two patients with grade 3, no grade 4).

#### 2.1.5. Anti-EGFR and Anti-MET Drugs: Combination of Lazertinib and Amivantamab

A widely studied possible option for the first-line treatment of patients with EGFR-mutated NSCLC is the combination of amivantamab and lazertinib, designed to enhance the efficacy of third-generation TKIs. Specifically, amivantamab, an EGFR-MET bispecific fully human antibody, can inhibit these two surface cell receptors and strengthens the innate immune system by promoting ADCC (antibody-dependent cellular cytotoxicity) and Fc-dependent trogocytosis. The action of amivantamab on MET enables it to work on cell clones whose resistance is dependent on MET activation [55]. In the phase III MARIPOSA trial, 1074 patients were divided into three arms: lazertinib, osimertinib, and amivantamab–lazertinib [56]. With a median PFS of 23.7 versus 16.6 months and HR of 0.70 (95% CI, 0.58–0.85), the experimental combination arm’s median PFS was considerably better than osimertinib. In patients with or without CNS metastases, the PFS benefit remained constant. Both arms’ ORRs were comparable, at 86% for amivantamab–lazertinib and 85% for osimertinib. Amivantamab–lazertinib was found to be effective in high-risk subgroups, particularly in patients with liver metastases at baseline (median PFS was 18.2 against 11.0 months) and TP53 co-mutations (median PFS was 18.2 versus 12.9 months for osimertinib), according to a secondary analysis from this research [57]. Furthermore, in patients who had detectable circulating tumor DNA (ctDNA) at baseline (HR 0.68 [0.53–0.86]) and in patients who did not clear ctDNA at C3D1 (0.48 [0.27–0.86]), amivantamab–lazertinib prolonged PFS. During the European Lung Cancer Congress 2025, data related to OS and icPFS were shown. Amivantamab+lazertinib showed a statistically significant and clinically relevant improvement in OS compared to osimertinib at a median follow-up of 37.8 months (HR for death, 0.75; 95% CI, 0.61–0.92; *p* < 0.005). The amivantamab+lazertinib arm’s median OS was not estimable (NE; 95% CI, 42.9–NE), while that of the osimertinib arm was 36.7 months (95% CI, 33.4–41.0). It is predicted that amivantamab+lazertinib will extend median OS by at least 12 months in comparison to osimertinib, assuming an exponential distribution of OS in both arms. Compared to 51% of patients in the osimertinib arm, 60% of patients in the amivantamab+lazertinib arm were still alive after 36 months. Amivantamab + lazertinib demonstrated a clinically meaningful improvement in icPFS with durable responses: the 3-year landmark icPFS was 36% vs. 18% for amivantamab + lazertinib vs. osimertinib [58]. The amivantamab–lazertinib combination has non-negligible toxicity, which is a safety concern [56]. Compared to 43% of patients treated with osimertinib, 75% of patients treated with amivantamab–lazertinib experienced grade ≥ 3 side effects, including infusion-related reactions (IRRs), skin rashes, and toxicities of the nails. In the experimental arm, 59% of patients and 35% of patients had, respectively, a dose reduction or a discontinuation of the drug, compared to 5% and 14% with osimertinib. Amivantamab–lazertinib dramatically raised the incidence of venous thromboembolic events (VTEs) by up to 37% compared to 9% in the control group, particularly in the four months after treatment started. As a result, it was advised to prescribe systematic prophylactic anticoagulation.

#### 2.1.6. Role of Radiotherapy

Local ablative treatments, and in particular radiotherapy, may also play a role in the first-line treatment of patients with EGFR-mutated NSCLC, both to treat any residual disease after a good partial response to TKIs, and to be used upfront together with TKIs, in case of oligometastatic disease. For this second objective, the phase II SINDAS study was designed, which evaluated the addition of stereotactic body radiation therapy (SBRT) to first-generation TKIs in first-line therapy, demonstrating that the incorporation of upfront local therapy via radiotherapy, in contrast to the use of a first-line TKI alone, resulted in a statistically significant enhancement in PFS (20.2 months versus 12.5 months (*p* < 0.001) and OS (25.5 months versus 17.4 months (*p* < 0.001) for patients with EGFR-mutated NSCLC [59]. In the TKI with RT arm, treatment resulted in a 6% incidence of symptomatic grade 3–4 pneumonitis and no grade 5 occurrences. On this topic, the results of the phase II NORTHSTAR study are expected, in which local ablative therapies such as surgery or radiotherapy are evaluated on residual disease in combination with osimertinib, after 6–12 weeks of TKIs [60].

### 2.2. Strengthening Second-Line Treatment

Following the development of progression after first-line treatment, it becomes essential to search for the resistance mechanisms responsible for progression, to guide subsequent therapeutic choices. In case of oligoprogression, corresponding to the appearance or evolution of tumor lesions at a few limited locations, that can be treated with local therapy, such as radiotherapy or surgery, and the indication to continue TKIs beyond progression is widely shared [61,62]. The results of the phase II LAT-FLOSI and phase II/III HALT studies should help to evaluate treatment with SBRT as a new standard option, in cases of oligoprogression under EGFR TKIs [63,64]. In case of systemic progression, the current standard of care is represented by platinum- and pemetrexed-based chemotherapy, even if with a limited efficacy [65]. In fact, platinum–pemetrexed yields an ORR of 27–42.9% and median PFS of 4.4–5.6 months. A tissue re-biopsy and/or liquid biopsy are necessary for the molecular analysis of the disease to comprehend the biological reasoning behind its progression and to plan the subsequent course of treatment.

#### 2.2.1. Combination of Anti-EGFR and Anti-MET Drugs in the Second-Line Treatment

The finding in 10–15% of cases of alterations in the MET gene, in particular amplification, has led to the development of several studies based on the combination of anti-EGFR and anti-MET drugs (Table 3). In the phase Ib TATTON trial, which included not only patients with MET amplification but also with MET overexpression, the combination of osimertinib and savolitinib, an oral, potent, and highly selective MET TKI, produced a median PFS of 5.5 months and an ORR of 33% [66,67]. The phase II ORCHARD trial, designed to identify the mechanisms underlying first-line osimertinib resistance and investigate strategies for overcoming acquired resistance, demonstrated, in the subgroup of 20 patients with MET alterations, the efficacy of the association of osimertinib–savolitinib [68]. The significance of MET status was further demonstrated by the ongoing phase II SAVANNAH’s interim results [69]. MET overexpression (IHC 3+) found in ≥90% of tumor cells and/or MET copy number ≥10 (fluorescence in situ hybridization—FISH) were used to identify the high MET-expressing group (34% of the population). The median PFS for this subgroup was 7.1 months, and the ORR was 49%. By contrast, there was no indication of efficacy in the remaining sample (ORR 9%, mPFS 2.8 months). Based on the outcome of the previously mentioned studies, the phase III SAFFRON study is currently comparing osimertinib plus savolitinib versus conventional chemotherapy, in patients with overexpressed and/or amplified MET. In INSIGHT 2, a phase II trial for patients with MET amplification following osimertinib as first-line therapy, 128 patients received oral tepotinib plus osimertinib once daily [70]. The ORR was 50.0%, the median OS was 17.8 months, the median PFS was 5.6 months, while the mDoR was 8.5 months. With an 8.3% ORR, tepotinib monotherapy had modest antitumor efficacy, supporting the advantages of EGFR and MET combination inhibition. This result implies that, rather than focusing only on MET, managing both oncogenic drivers is essential after NSCLC cells develop osimertinib resistance through MET amplification. Peripheral oedema, decreased appetite, extended electrocardiogram QT interval, and pneumonitis were the most frequent treatment-related grade 3 or worse side effects. Specifically, 13% of patients experienced serious treatment-related side events.

#### 2.2.2. Combination of Lazertinib and Amivantamab in the Second-Line Treatment

With bispecific antibodies, the possibility of acting on both EGFR and MET is exploited. The lazertinib–amivantamab combination was originally tested in the second-line treatment. In the CHRYSALIS trial, specifically in cohort E, an ORR of 36% and a median PFS of 4.9 months were reached, with a better response in patients with high EGFR/MET expression [71]. In the CHRYSALIS2 trial, patients with MET overexpression achieved an ORR of 61% vs. 14% of patients without MET overexpression [72,73] The results of these studies led to the design of MARIPOSA-2, a phase III international trial. In this study, 657 patients with disease progression during therapy with osimertinib as the most recent line of treatment were included [74]. When compared to CT alone (median PFS of 4.2 months), PFS was considerably higher in both experimental arms, with a median PFS of 8.3 months for amivantamab–lazertinib plus CT (A-L-CT) and 6.3 months for amivantamab plus CT (A-CT). The ORR was 64% for A-CT, 63% for A-L-CT, and 36% for CT. When compared to chemotherapy alone (8.3 months), intracranial PFS improved similarly in both experimental arms (median of 12.5 months). OS data are not available yet. In both experimental arms, the number of grade ≥3 adverse events (AEs) increased significantly, reaching 92% (A-L-CT), 72% (A-CT), and 48% (CT). In 34% of cases (A-L-CT), 18% of cases (A-CT), and 4% of cases (CT), treatment was discontinued permanently. In the regimens comprising amivantamab, hematologic toxicities (including neutropenia, thrombocytopenia, anemia, and leucopenia) were the most common adverse events. Additionally, 58% and 56% of patients receiving A-CT and A-L-CT, respectively, experienced infusion-related reactions (IRRs), whose incidence can be reduced with adequate steroid-based premedication. Even in the subcutaneous (SC) formulation, amivantamab has been shown to be effective in the phase III PALOMA-3 trial, in association with lazertinib [75]. When compared to intravenous (IV) amivantamab, SC treatment demonstrated similar pharmacokinetics and a noninferior ORR (30.1%), with a positive trend in PFS (6.1 vs. 4.3 months), a longer median DOR (11.2 vs. 8.3 months for IV) and a better OS (HR 0.62 [0.42–0.92]). With SC amivantamab, lower IRR (13% vs. 66% with IV) and VTE rates (9% vs. 14% with IV) were recorded.

#### 2.2.3. Combination of Anti-EGFR and Other Target Therapies

While the combination of dabrafenib and trametinib is recommended in the treatment of BRAF V600E-mutated NSCLC in the main guidelines, in the event of the appearance of a BRAFV600E mutation as a resistance mechanism after osimertinib progression, there is currently disagreement over the best course of action for concurrently addressing BRAF and EGFR in lung cancer. Case reports have been reported in the literature with evidence of the efficacy of the association between osimertinib and anti-BRAF therapy [76,77,78]. In particular, in a phase 2 clinical trial, patients with BRAF V600E-mutant lung cancer were found to benefit clinically from vemurafenib, a BRAF inhibitor authorized for the treatment of BRAF V600E-mutant melanoma [79].

In the second-line context, ALK rearrangement as an osimertinib resistance mechanism is uncommon (<1%). There are very few case studies in the literature on suitable treatment approaches for these situations [80,81,82]. Further studies are required to provide evidence of the combination’s efficacy, safety, and optimal administration method (concurrent vs. alternative).

#### 2.2.4. Role of Antibody-Drug Conjugates (ADCs)

There is considerable interest in exploring the role of ADCs in NSCLC with actionable genomic alterations after failure of traditional treatments. The increased human epidermal growth factor receptor 3 (HER3) signaling in NSCLC patients who become resistant to EGFR TKIs has been an interesting finding [83]. Research has consistently demonstrated that elevated HER3 expression is linked to more aggressive NSCLC with worse outcomes, indicating its potential as a prognostic marker. Also, overexpression of heregulin (HRG), HER3’s ligand, adds to the disease’s aggressiveness, by increasing tumor proliferation and metastatic potential. HRG-mediated HER3 activation results in downstream PI3K/AKT pathway signaling, which enhances cell survival and confers resistance to EGFR-targeted treatments. Patritumab–deruxtecan is an ADC in which a human monoclonal antibody directed against HER3 is linked to deruxtecan, a topoisomerase I inhibitor. In the HERTHENA-lung01 phase II trial, 275 patients with EGFR common mutations who had previously had platinum-based chemotherapy and EGFR TKIs received patritumab–deruxtecan at a dose of 5.6 mg/kg every three weeks [84]. A median OS of 11.9 months, a median PFS of 5.5 months, and an ORR of 29% have been reported. Furthermore, in 30 patients with brain metastases who had not received previous radiation therapy, the intracranial ORR was 33%. Forty-five percent of patients experienced grade ≥ 3 adverse events, with hematological toxicity and interstitial lung disease (ILD) described as the most frequently reported adverse events. The phase III HERTHENA-Lung02 study will provide further information on the role of patritumab–deruxtecan compared to standard chemotherapy after TKI failure [85]. A further promising ADC is datopotamab–deruxtecan (Dato-Dxd), in which a trophoblast cell surface antigen 2 (TROP2) directed monoclonal antibody is linked to a topoisomerase I inhibitor payload via a cleavable linker. In the phase III TROPION-Lung01 trial, Dato-DXd dramatically increased PFS compared to docetaxel in patients with advanced or metastatic NSCLC, in patients with non-squamous histology, and, in particular, with actionable genomic alterations (AGAs) [86]. The TROPION-Lung05 phase II trial evaluated the safety and clinical activity of Dato-DXd at a dose of 6 mg/kg once every 3 weeks in patients with advanced/metastatic NSCLC with AGAs progressing during or after targeted therapy and platinum-based chemotherapy [87]. Overall, of 137 treated patients, 56.9% had EGFR mutations. In this population, the confirmed ORR was 43.6% (95% CI, 32.4 to 55.3), the median DOR was 7.0 months (95% CI, 4.2 to 9.8), the overall DCR was 82.1% (95% CI, 71.7 to 89.8), and the median PFS was 5.8 months (95% CI, 5.4 to 8.3). Additionally, 28.5% of patients experienced treatment-related adverse events (TRAEs) of grade ≥3, with stomatitis reported as the most prevalent TRAEs. Five patients (3.6%) developed ILD/pneumonitis as a result of the treatment, and one patient (0.7%) sustained a grade 5 AE.

#### 2.2.5. Role of Immunotherapy

The role of immunotherapy after progression to TKIs in patients with EGFR-mutated NSCLC is still somewhat controversial [88]. The phosphorylated extracellular signal-regulated kinases 1/2/phosphorylated c-Jun (p-ERK1/2/p-c-Jun) signaling pathway and inhibition of Nuclear Factor kappa-light-chain-enhancer of activated B cells (NF-κB) are two mechanisms through which EGFR activation appears to induce programmed death-ligand 1 (PD-L1) expression, resulting in T-cell apoptosis and immune response suppression [89,90]. A pooled analysis of 15 studies revealed that PD-L1 expression was lower in EGFR-mutant NSCLCs [91]. Furthermore, EGFR mutations were linked to a lower tumor mutational burden (TMB) in comparison to wild-type EGFR, offering a scientific justification and a possible explanation for this subgroup’s poor immunotherapy response [92]. Several prospective and retrospective studies have failed to demonstrate a significant benefit of immunotherapy as a single agent compared with chemotherapy in previously treated EGFR-mutated NSCLC [88]. Numerous studies have assessed the possible advantages of a combination with chemotherapy in EGFR-mutated NSCLC. Nivolumab + chemotherapy did not significantly enhance PFS compared to chemotherapy alone, according to CheckMate 722 (median PFS 5.6 vs. 5.4 months, HR 0.75, *p* = 0.0528) [93]. In TKI-resistant EGFR-mutant metastatic non-squamous NSCLC, the most recent phase III data from the KEYNOTE-789 trial, evaluating the safety and effectiveness of combining pembrolizumab with chemotherapy, have demonstrated that pembrolizumab with chemotherapy did not significantly enhance PFS or OS over chemotherapy alone [94]. In patients with EGFR-mutated NSCLC, the IMpower 150 study showed encouraging efficacy for a combination of immune chemotherapy and bevacizumab [95]. In 22 patients with EGFR-mutated NSCLC after TKI failure, an open-label, single-arm, phase II trial assessed the immune cell profile and effectiveness of the modified regimen consisting of atezolizumab, bevacizumab (7.5 mg/kg), and chemotherapy [96]. The DCR was 100% while the ORR was 42.9%, with 6.3 months of median PFS. The ORR (75 vs. 23.1%; *p* = 0.032) and PFS (14.0 vs. 6.1 months; *p* = 0.022) were substantially greater in patients with PD-L1 expression ≥1% than in those with PD-L1 expression <1%. The phase III ORIENT-31 trial compared sintilimab, an antibody anti-PD1, plus the bevacizumab biosimilar IBI305 plus pemetrexed–cisplatin versus sintilimab plus pemetrexed–cisplatin or chemotherapy alone, in pretreated patients with a sensitizing EGFR mutation [97]. When compared to chemotherapy alone, sintilimab plus chemotherapy increased PFS (median 5.5 months [95% CI 4.5–6.1] vs. 4.3 months [4.1–5.3]), and sintilimab plus IBI305 plus chemotherapy maintained a significant PFS benefit (median 7.2 months [95% CI 6.6–9.3). In contrast to 19.2 months for chemotherapy alone, the median OS was 21.1 months (95% CI 17.5–23.9) for the sintilimab-plus-IBI305-plus-chemotherapy group and 20.5 months for the sintilimab-plus-chemotherapy group. Regarding safety, treatment-related adverse events of grade 3 or worse happened in 56% of patients in the sintilimab-plus-IBI305-plus-chemotherapy group, 41% of patients in the sintilimab-plus-chemotherapy group, and 49% of patients in the chemotherapy-alone group. In this area, encouraging data have been derived from studies evaluating ivonescimab, a bispecific antibody targeting both PD-1 and VEGF. In patients whose disease progresses following EGFR-TKI treatment, the HARMONi-A trial, a double-blind, placebo-controlled, randomized phase III study, compares the effectiveness of ivonescimab with chemotherapy versus chemotherapy alone [98]. When ivonescimab was added to chemotherapy, the primary endpoint, PFS, improved significantly, with a median of 7.1 months compared to 4.8 months for the placebo (HR, 0.46 [95% CI, 0.34–0.62]; *p* < 0.001). Patients with brain metastases and those subjected to osimertinib also benefited from PFS. Data on survival are not yet mature. These findings support earlier research indicating that VEGF suppression is necessary for anti-PD(L)-1 and chemotherapy to be beneficial in EGFR-mutated patients.

## 3. Conclusions

Almost two decades have passed since the discovery of the first EGFR mutations in NSCLC. As understanding has grown throughout the years, the natural history of this disease has evolved. Specifically, there have been numerous developments from a therapeutic and diagnostic perspective. Regarding the diagnosis, next-generation sequencing technology allows the simultaneous testing of numerous predictive and prognostic biomarkers with high sensitivity and specificity. Specifically, it enables the identification of multiple EGFR mutations and other oncogenic molecular targets; it also makes it possible to identify the mechanisms of resistance that may arise during therapy, guiding subsequent therapeutic choices. In terms of therapeutic advancements, the clinical history of EGFR-mutated NSCLC has been completely transformed by the introduction of TKIs. To overcome resistance mechanisms and improve survival and quality of life, novel TKIs with increased selectivity and greater blood–brain barrier penetration have been investigated. Notwithstanding the good responses recorded, most patients undergo disease progression. The emergence of resistance mechanisms has led to the search for new therapeutic strategies to improve the prognosis of patients. In this literature review, we have reported the main studies designed with the aim of developing therapeutic strategies to improve the prognosis of EGFR-mutated NSCLC. Despite the achieved results, there are several critical points to consider. Specifically, if it is true that improving the first line of treatment leads to greater results, this presents a toxicity issue because, often, there is also an increase in adverse effects. The recently announced data, in particular, about the combination of amivantamab–lazertinib and osimertinib–CT in addition to standard osimertinib, have the potential to revolutionize clinical practice. Due to the increased risk of side effects, combination therapies are undoubtedly not suitable for every patient and must be customized based on the individual’s features. Osimertinib monotherapy may still be beneficial for patients who are elderly, have a modest disease burden, or have a poor performance status (PS). Conversely, combinations of osimertinib–CT or amivantamab–TKIs can be used in younger patients, with a good PS or with a higher burden of disease or in the presence of prognostic commutations of greater aggressiveness. Increased toxicity is by no means a minor issue. Also, the use of antiangiogenic drugs, like bevacizumab or ivonescimab, in patients who are more likely to experience bleeding is also limited by the possibility of increased adverse effects, such as bleeding or thromboembolic events, hypertension, or renal toxicity. The introduction of new drugs, such as ADCs or bispecific antibodies, has also brought with it the need to deal with new side effects, different from those known related to drugs used in clinical practice. Additionally, once first-line combination therapies are employed, the restricted availability of drugs for succeeding lines is another crucial factor. Although novel medications like ADCs have shown promising outcomes, their position in the therapeutic algorithm is still unclear, and further research is required to fully realize their potential. Moreover, comparing different studies is quite challenging because of the significant variables involved. In conclusion, for patients with EGFR-mutated NSCLC, there is a plethora of therapeutic options, and many more could arrive in the future, both in the first line and in the subsequent lines. It is therefore essential to be guided by the molecular re-evaluation of the disease, through a tissue biopsy or liquid biopsy, to identify the resistance mechanism and then choose a targeted therapy or propose enrollment in clinical trials.

## Figures and Tables

**Table 1:** Main clinical studies of combination of TKIs and chemotherapy.

Clinical Trial	Treat Ments	PTS (*n*)	ORR	Median icPFS	Median PFS	Median OS
GAP-Brain [33]	Gefitinib + ChT vs. Gefitinib	161	80.0% (95% CI, 71.0–89.0%) vs. 64.2% (95% CI, 53.5–74.9%)	15.6 mo (95% CI, 14.3–16.9) vs. 9.1 mo (95% CI, 8.0–10.2)	16.3 (95% CI, 14.4–18.2) vs. 9.5 mo (95% CI, 8.3–10.8)	35.0 vs. 28.9 mo; hazard ratio, 0.65; 95% CI, 0.43–0.99
NEJ009 [31]	Gefitinib + ChT vs. Gefitinib	345	-	-	20.9 (95% CI, 18.0–24.0) vs. 18.0 mo (95% CI, 16.3–20.7)	49.03 (95% CI, 41.77–56.73) vs. 38.47 mo (95% CI, 31.1–47.1)
Noronha et al. [32]	Gefitinib + ChT vs. Gefitinib	350	-	-	16 (95% CI, 13.5 to 18.5) vs. 8 mo (95% CI, 7.0–9.0)	not reached vs. 17 mo (95% CI, 13.5–20.5)
FLAURA-2 [34]	Osimertinib + ChT vs. Osimertinib	557	83% (95% CI, 78–87) vs. 76% (95% CI, 70–80)	-	25.5 (95% CI, 24.7–NC) vs. 16.7 mo (95% CI, 14.1–21.3)	-

PTS: patients; n: number; ChT: chemotherapy; mo: months; ORR: overall response rate; icPFS: intracranial progression-free survival; PFS: progression-free survival; OS: overall survival.

**Table 3:** Main clinical studies of combination of anti-EGFR and anti-MET agents.

Clinical Trial	Phase	ORR	Median DoR	Median PFS	Median OS
TATTON [63]	1b	33%	-	5.5 months	-
ORCHARD [64]	2	-	-	-	-
SAVANNAH [65]	2b	49%	-	7.1 months	-
INSIGHT2 [66]	2	50%	8.5 months	5.6 months	17.8 months

ORR: overall response rate; DoR: duration of response; PFS: progression-free survival; OS: overall survival.
